# Prediction and Analysis of the Residual Capacity of Concrete-Filled Steel Tube Stub Columns under Axial Compression Subjected to Combined Freeze–Thaw Cycles and Acid Rain Corrosion

**DOI:** 10.3390/ma12193070

**Published:** 2019-09-20

**Authors:** Tong Zhang, Xuetao Lyu, Yang Yu

**Affiliations:** 1 School of Civil Engineering, Liaoning Technical University, Fuxin 123000, China; 2 School of Resource and Civil Engineering, Liaoning Institute of Science and Technology, Benxi 117004, China; 3 Transportation and Civil Buildings College, Foshan University, Foshan 528225, China; 4 Shaanxi Key Laboratory of Safety and Durability of Concrete Structures, Xi’an 710123, China; 5 School of Civil and Environmental Engineering, University of Technology Sydney, Sydney, NSW 2007, Australia

**Keywords:** Concrete-filled steel tube stub column, combined freeze–thaw cycle and acid rain corrosion, residual capacity, finite element analysis

## Abstract

This paper presents a theoretical investigation on the safety evaluation, stability evaluation, and service life prediction of concrete-filled steel tube (CFST) structures in a Northern China area with acid rain. The finite element software ABAQUS was used to establish a numerical model, which was used to simulate the axial compression behavior of CFST columns subjected to the combined actions of freeze–thaw cycles and acid rain corrosion. The model performance was validated using the experimental results of the evaluation of mechanical properties, including the failure mode and load–displacement curve. Then, the effects of the section size, material strength, steel ratio, and combined times on the residual capacity were studied. The results show that the section size has a smaller influence on the residual strength than the other parameters and can be neglected in the design procedure. However, the other parameters, including the material strength, steel ratio, and combined times have relatively large influences on the axial compressive performance of CFST stub columns subjected to a combination of freeze–thaw cycles and acid rain corrosion. Finally, design formulae for predicting the residual strength of CFST stub columns that are under axial compression and the combined effect of freeze–thaw cycles and acid rain corrosion are proposed, and their results agree well with the numerical results.

## 1. Introduction

Concrete-filled steel tube (CFST) structures are widely used in real engineering applications in many countries because of their high bearing capacity and good seismic performance [1,2,3,4,5,6,7]. With the rapid development of research and applications, many countries worldwide have promulgated technical specifications for CFST structures [8,9,10,11,12,13]. To date, the main research in this area has focused on the static performance [14,15,16,17], seismic performance [18,19,20], and fire resistance [21,22] of CFST structures, including studies on their structural durability after acid rain corrosion [23] and freeze–thaw cycles [24]. However, previous research on the durability of CFST structures has been limited to a single environmental factor. Few studies have been conducted on the effects of dual or multiple environmental factors on the mechanical properties of CFST structures. For instance, combined changes in positive and negative temperatures are frequent in Northern China, and structures often suffer from frost damage due to low temperatures in winter [25,26,27]. Additionally, acid rain pollution in summer is becoming increasingly serious in the coastal areas, and it can result in the corrosion of steel structures [28,29]. These problems have greatly affected the mechanical properties of CFST structures that experience freeze–thaw cycles and acid rain corrosion, as well as other environmental factors. 

This study aims to develop a theoretical solution to the above problems related to the safety evaluation, stability evaluation, and service life prediction of CFST structures subjected to acid rain in Northern China. First, squared CFST (SCFST) stub columns were selected as the research object. On the basis of modifications to the ultimate stress of the core concrete and the yield strength of the external steel tube, the mechanical behaviors of structures subjected to the combined action of freeze–thaw cycles and acid rain corrosion were analyzed by finite element (FE) modeling. Then, the stress mechanism and parameters that affect the residual bearing capacity, together with the corresponding influence rule, were studied. The outcomes of this research can provide theoretical guidance for the design of CFST structures located in areas with acid rain, which is of great significance to practical engineering applications.

The rest of this paper is organized as follows. Section 2 develops numerical models, and their performances are validated using experimental results from the published literature. In Section 3, the whole process of the load–displacement relationship of the specimen during axial compression is analyzed, including the analyses of the failure mode, load–displacement curve, and bearing capacity. Section 4 analyzes the influences of different parameters on the bearing capacity of the specimen and proposes two design formulae as theoretical references for practical engineering applications. Finally, conclusions are drawn in Section 5.

## 2. Development and Validation of the Finite Element Model 

### 2.1. Stress–Strain Relationships of Materials

A square CFST column is generally composed of steel and concrete materials. The influence of acid rain includes the corrosion of the exterior steel tube, and corrosion is able to reduce not only the effective thickness but also the mechanical properties of the steel tube. In this study, the stress–strain relationship of the steel was adopted from [30], and this relationship accounts for effects due to the corrosion of the steel tube. Freezing and thawing effects can cause the core concrete to become frozen, which can cause internal cracks and weaken the strength of the column. Accordingly, the uniaxial stress–strain relationship model of core concrete from [24], which considers the effect of freeze–thaw cycles, was used in this study. The relevant mathematical expressions are provided below.

(1) The Stress–Strain Relationship Model of Steel:

(1) $$\sigma_{s}=\left\{\begin{array}{c} E_{se}\varepsilon_{s},\;\;\;\;\;\;\;\;\;\;\;\;\;\;\;\;\;\;\;\;\varepsilon_{s}\leq \varepsilon_{e}\\ -A\varepsilon_{s}^{2}+B\varepsilon_{s}+C,\;\;\;\;\;\;\;\;\varepsilon_{e}<\varepsilon_{s}\leq \varepsilon_{e1}\\ f_{ye},\;\;\;\;\;\;\;\;\;\;\;\;\;\;\;\;\varepsilon_{e1}<\varepsilon_{s}\leq \varepsilon_{e2}\\ f_{ye}\left[1+0.6\frac{\varepsilon_{s}-\varepsilon_{e2}}{\varepsilon_{e3}-\varepsilon_{e2}}\right],\;\;\;\;\varepsilon_{e2}<\varepsilon_{s}\leq \varepsilon_{e3}\\ 1.6f_{ye},\;\;\;\;\;\;\;\;\;\;\;\;\;\;\;\;\;\;\varepsilon_{s}>\varepsilon_{e3} \end{array}\right.$$

*ε*_e_ = 0.8*f*_ye_/*E*_se_ (2)

*ε*_e1_ = 1.5*ε*_e_ (3)

*ε*_e2_ = 10*ε*_e1_ (4)

*ε*_e3_ = 100*ε*_e1_ (5)

*A* = 0.2*f*_y_(*ε*_e1_−*ε*_e_)^2^ (6)

*B* = 2*Aε*_e1_ (7)

*C* = 0.8*f*_ye_+*Aε*_e_^2^ − *Bε*_e_ (8)

*E*_se_ = (1 − 0.955*γ*)*E*_s_ (9)

*f*_ye_ = (1 − 1.007*γ*)*f*_y_ (10)

*γ* = ∆*t/t* (11)

∆*t = t − t_e_* (12)

where *E_s_* denotes the initial elastic modulus of steel, *E_se_* denotes the effective elastic modulus of corroded steel, *f_y_* denotes the initial yield strength of steel, *f_ye_* denotes the effective yield strength of corroded steel, *γ* denotes the corrosion rate, ∆*t* denotes the decreasing value of the wall thickness of the corroded steel tube, *t* denotes the initial wall thickness of the steel tube, and *t_e_* denotes the effective wall thickness of the corroded steel tube.

**Figure 1:**
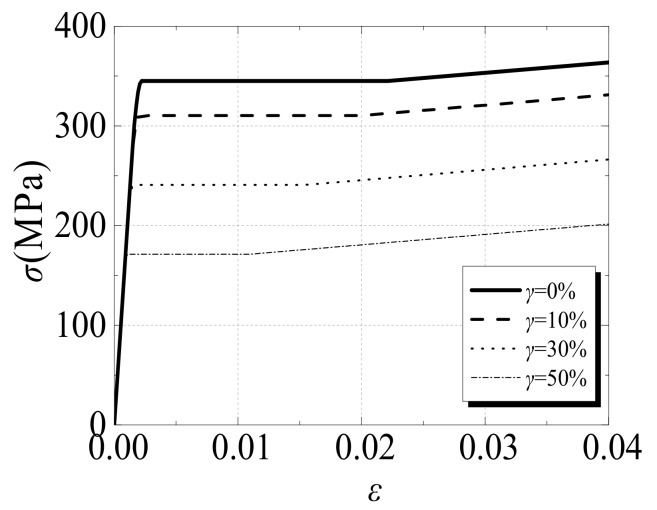
The stress–strain relationship of a steel tube after acid rain corrosion.

Figure 1 shows the stress–strain relationship of a typical steel tube after it is subjected to acid rain corrosion, as calculated by the model proposed above. The related parameter setting for the numerical simulation is *f*_y_ = 345 MPa, and the range of *γ* is 0–50%.

(2) The Stress–Strain Relationship Model of Concrete:

(13) $$y=\left\{\begin{array}{c} 2x-x^{2},\;\;\;\;\;\;\;x\leq 1\\ \frac{x}{\beta {\left(x-1\right)}^{\eta }+x},\;\;x>1 \end{array}\right.$$

(14) $$x=\varepsilon /\varepsilon_{0}$$

(15) $$y=\sigma /\sigma_{0}$$

(16) $$\sigma_{0}={f^{\prime }}_{c}\left(1-0.065N_{\mathrm{ft}}/100\right)$$

(17) $$\varepsilon_{0}=\left(1300+12.5{f^{\prime }}_{c}+800\zeta_{e}^{0.2}\right)\times 10^{-6}$$

(18) η=1.6+1.5/x

(19) $$\beta ={\left({f^{\prime }}_{c}\right)}^{0.1}/\left[1.2\sqrt{1+\zeta_{e}}\right]$$

(20) $${f^{\prime }}_{c}=\left[0.76+0.2+\log_{10}\left(\frac{f_{cu}}{19.6}\right)\right]f_{\mathrm{cu}}$$

(21) $$\zeta_{e}=\alpha_{e}\cdot f_{\mathrm{ye}}/f_{\mathrm{ck}}$$

(22) $$f_{\mathrm{ck}}=0.88\times 0.76\times f_{\mathrm{cu}}$$

(23) $$\alpha_{e}=A_{\mathrm{se}}/A_{c}$$

(24) $$E_{c}=4700\sqrt{{f^{\prime }}_{c}})$$

(25) $$\psi =\left\{\begin{array}{c} 56.3\left(1-\zeta_{e}\right),\;\;\zeta_{e}\leq 0.5\\ 6.672e^{\frac{7.4}{4.64+\zeta_{e}}},\;\;\zeta_{e}>0.5 \end{array}\right.$$

(26) $$K_{c}=\frac{5.5}{5+2{\left({f^{\prime }}_{c}\right)}^{0.075}}$$

(27) $$f_{b0}/f_{c0}=1.5{\left({f^{\prime }}_{c}\right)}^{-0.075}$$

where σ_0_ and ε_0_ denote the peak stress and peak strain of the stress–strain relationship of concrete, respectively; *f_c_*′ denotes the cylinder compressive strength of concrete; *f_cu_* denotes the cube compressive strength of concrete; *f_ck_* denotes the standard value of the axial compressive strength of concrete; *ξ_e_* denotes the effective restraint coefficient of CFST; *α_e_* denotes the effective steel ratio of the steel tube; *A_se_* denotes the effective area of the steel tube; *A_c_* denotes the area of a concrete section; and *N_ft_* denotes the number of freeze–thaw cycles.

**Figure 2:**
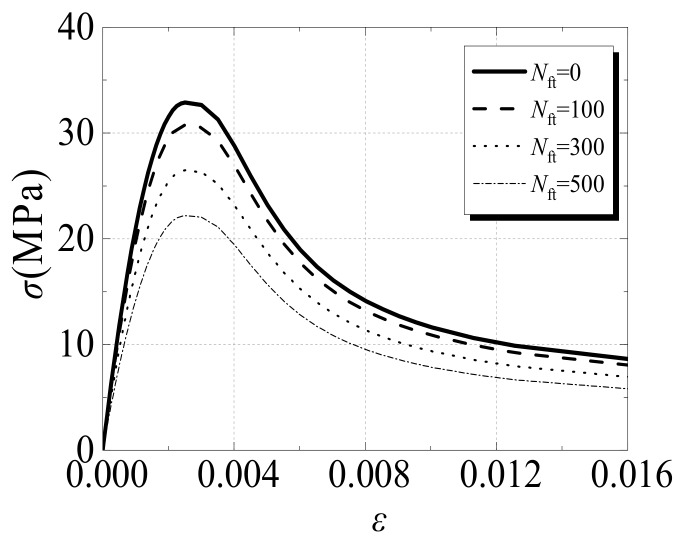
The stress–strain relationship of core concrete after freeze–thaw cycles.

Figure 2 shows the typical stress–strain relationship of core concrete after it is subjected to freeze–thaw cycles. The corresponding numerical parameters are *f_cu_* = 40 MPa, *ζ_e_* = 1.0, and the range of *N_ft_* is 0–500.

In this study, the Poisson’s ratio of concrete was set to 0.2. The elastic modulus of concrete (*E_c_*), dilation angle (*ψ*), the ratio of the second stress invariant on the tensile meridian to that on the compressive meridian (*K_c_*), and the ratio of the initial biaxial compressive yield stress to the initial uniaxial compressive yield stress (*f_b_*_0_/*f_c_*_0_) were set according to the corresponding parameter values in [31]. The relevant equations are

(28) $$E_{c}=4700\sqrt{{f^{\prime }}_{c}}$$

(29) $$\psi =\left\{\begin{array}{c} 56.3\left(1-\zeta_{e}\right),\;\;\zeta_{e}\leq 0.5\\ 6.672e^{\frac{7.4}{4.64+\zeta_{e}}},\;\;\zeta_{e}>0.5 \end{array}\right.$$

(30) $$K_{c}=\frac{5.5}{5+2{\left({f^{\prime }}_{c}\right)}^{0.075}}$$

(31) $$f_{b0}/f_{c0}=1.5{\left({f^{\prime }}_{c}\right)}^{-0.075}.$$

Furthermore, the eccentricity was set to 0.1, and the viscosity parameter was set to 0.0001 according to [32].

### 2.2. Numerical Modeling

**Figure 3:**
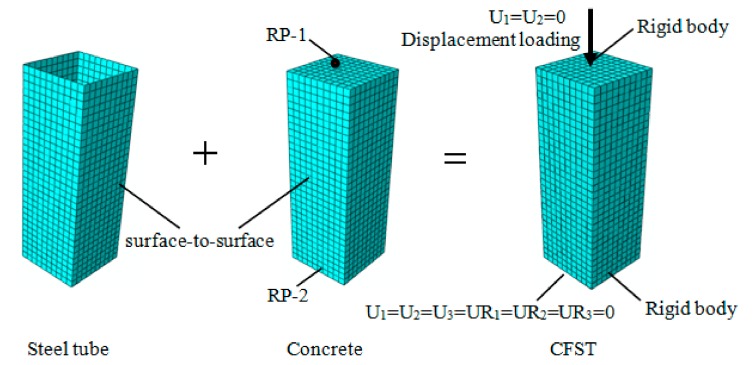
Finite element model meshing and boundary conditions.

In this numerical simulation, the FE software ABAQUS 6.13 was employed for modeling and analysis. The steel tube was simulated by the three-dimensional shell element S4R. The Simpson integral method with nine integral points was adopted in the thickness direction of the shell element to ensure calculation accuracy. Concrete was simulated by the three-dimensional solid element C3D8R. Surface-to-surface contact was utilized in the contact mode between the steel tube and concrete. Since the stiffness of the concrete is larger than that of the steel tube, the outer surface of the concrete was set to the main surface, and the inner surface of the steel tube was set to the slave surface. The contact between them was divided into the contacts in the normal direction and tangent direction. In the normal direction, this contact was designated as hard. The tangential friction coefficient was set to 0.6 according to [33]. In the simulation, there was no limit to the contact pressure on the surface between them. For the boundary conditions, one end of the column was set as a fixed constraint to constrain all six degrees of freedom. The other end was loaded with the displacement and only limited in its horizontal leveling ability, which is shown in Figure 3. It is clear that vertical displacement was applied in the U_3_-direction, with the other two degrees of freedom constrained. Both ends of the model were set as the rigid body to improve the calculation convergence and avoid stress concentration. The geometric center points RP-1 and RP-2 of both ends were taken as reference points. The end corresponding to each reference point was selected as the region type. The calculation cost and accuracy were balanced by taking the mesh size as 1/10 of the width of the cross-section of the column [34]. The meshing and boundary conditions of the FE model are also described in Figure 3.

### 2.3. Verification of the FE Model

The experimental results of the SCFST members from [24,30,35,36] subjected to the combined action of freeze–thaw cycles and acid rain corrosion were employed to validate the effectiveness of the developed FE model. The detailed analysis and discussion are presented in the following parts.

#### 2.3.1. Experimental Validation of the Freeze–Thaw Cycle Case

(1) Failure Mode

**Figure 4:**
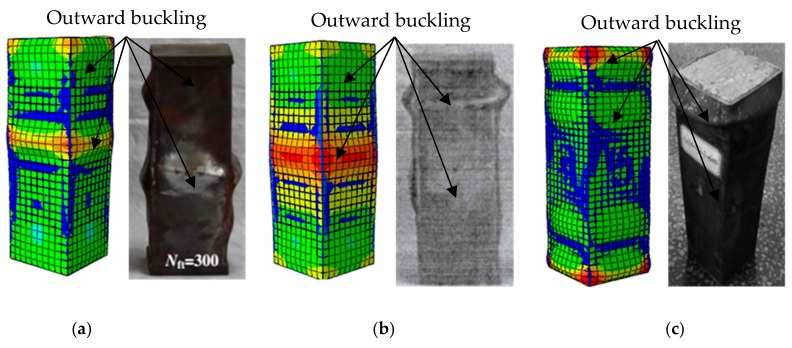
Comparison of failure modes between the numerical results and experimental results in the literature. (**a**) Sc1-300 specimen in [24]; (**b**) S2-100 specimen in [35]; and (**c**) SC30-T2.5-N100 specimen in [36].

Figure 4 compares the experimental results in [24,35,36] with the numerical results from the developed FE model. The subfigures show that the failure mode from the numerical analysis results agrees well with that from experimental observation under the same condition. In particular, outward buckling appeared at the end and center of the steel tube, which further validates the effectiveness of the proposed FE model in portraying the failure mode under the freeze–thaw cycle condition.

(2) Bearing Capacity

The numerical results of the axial bearing capacity of the specimens are provided in Table 1, in which *B* and *t* denote the width of the specimen section and the thickness of the steel tube, respectively. *N_ue_* denotes the experimental result of the bearing capacity of the specimen; *N_ce_* denotes the corresponding bearing capacity simulation result in the literature; and *N_be_* denotes the result of the bearing capacity of the specimen calculated by the FE model in this study. The comparison results reveal that the prediction errors of *N_ue_*/*N_be_* of almost all the specimens were within ±10%, except for the S3-100, S3-300, S3.9-200, and S3.9-300 specimens. Moreover, the mean value of *N_ue_*/*N_be_* was lower than that of *N_ue_*/*N_be_*; this finding indicates that numerical results of the FE model developed in this study are closer to the corresponding experimental results than the numerical results from the literature. Therefore, it can be concluded that the developed FE model can effectively predict the bearing capacity of SCFST members subjected to freeze–thaw cycles.

(3) Axial Load–Displacement (or Strain) Curve

**Figure 5:**
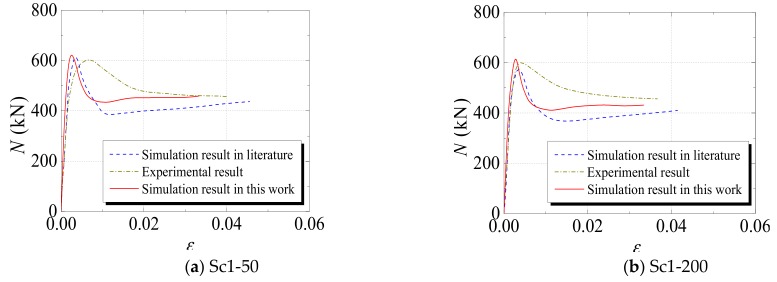

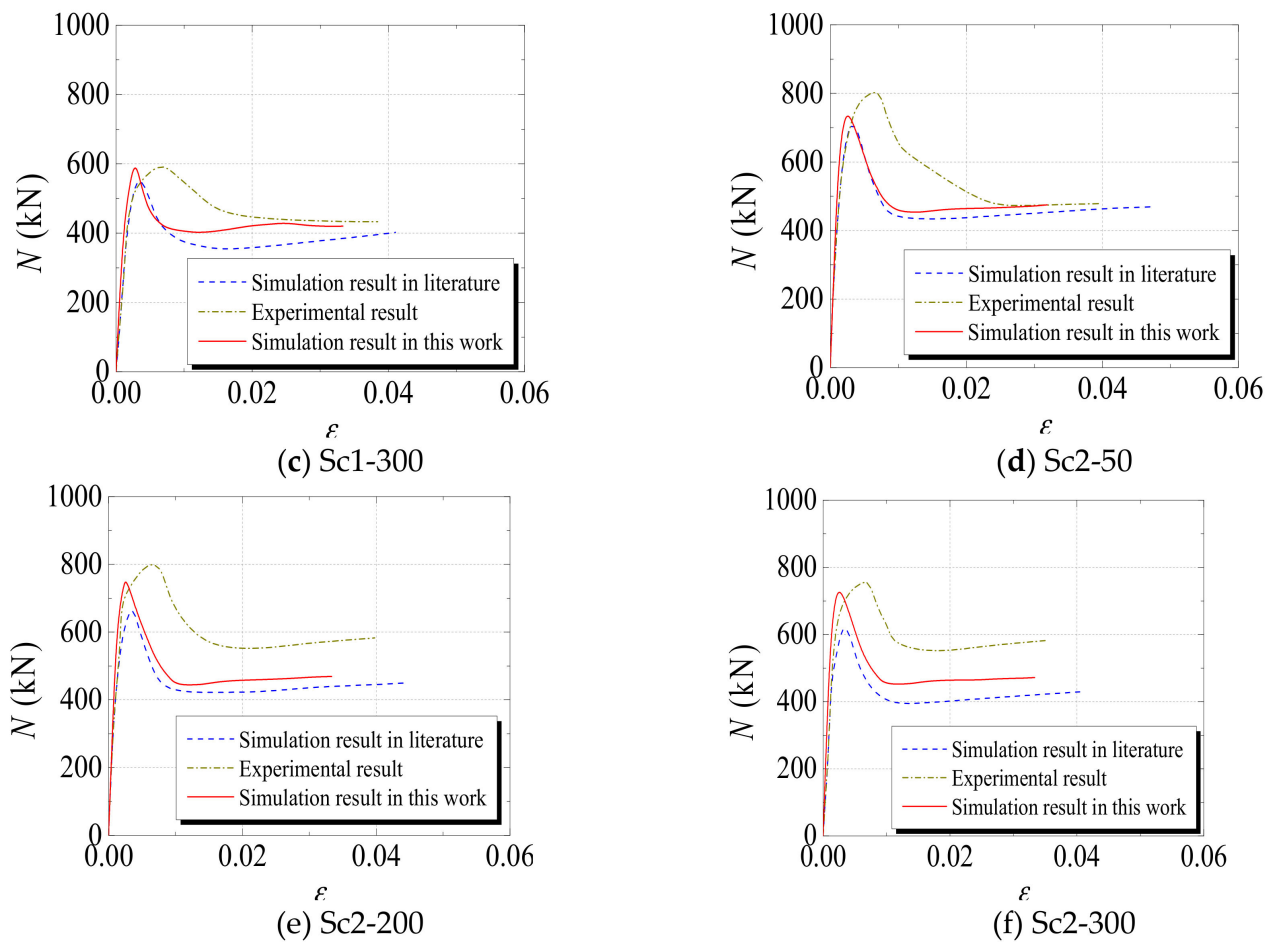
Comparisons between numerical results in this study and the results (both experimental and numerical) in [24].

**Figure 6:**
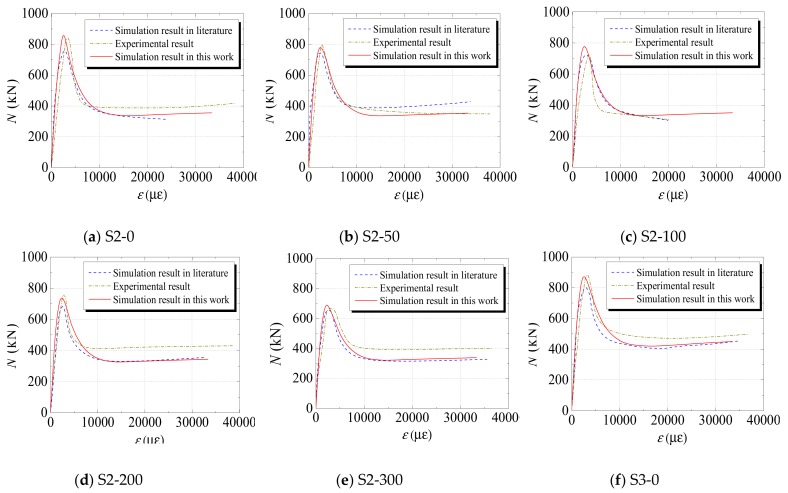

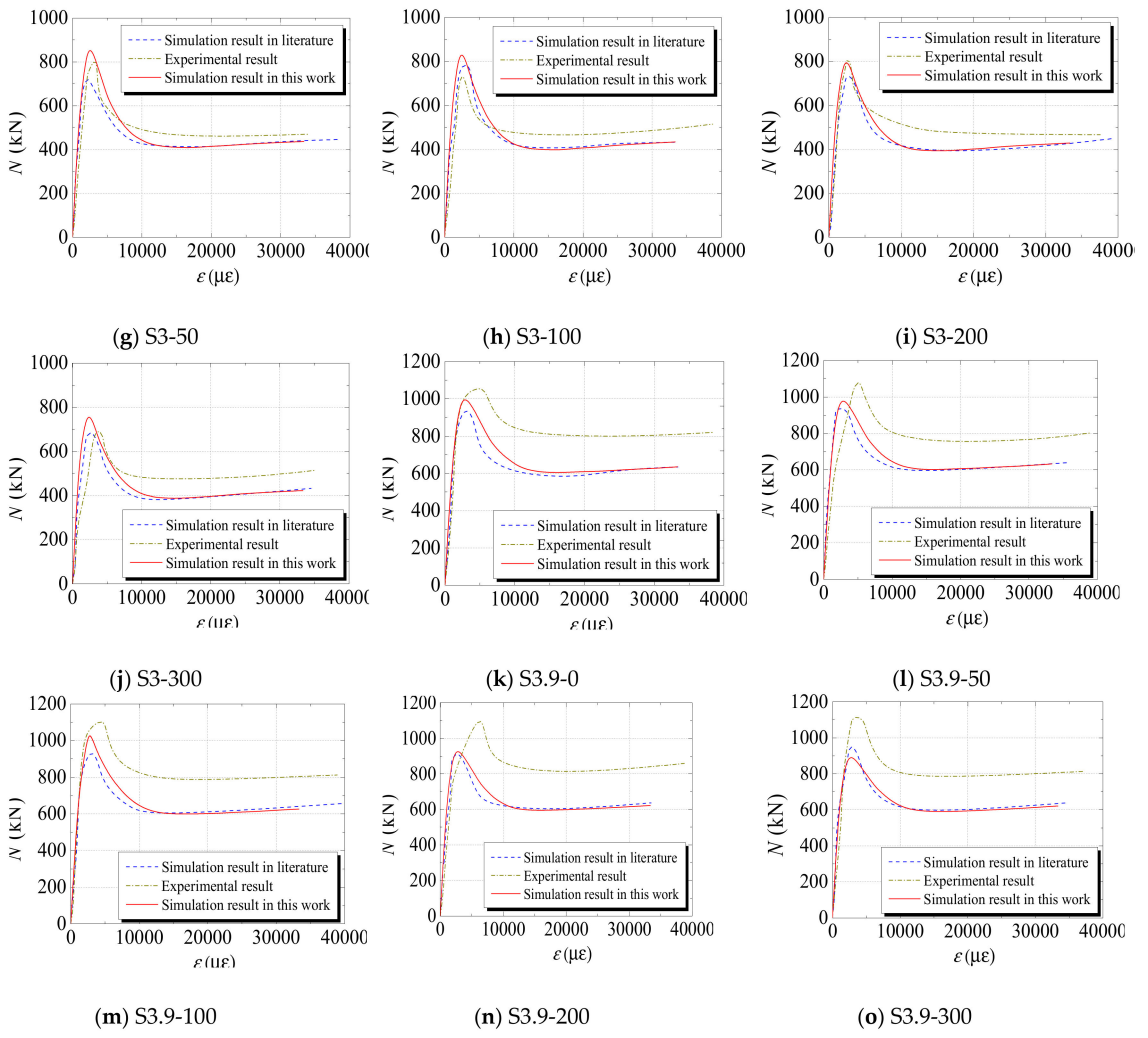
Comparisons between the numerical results in this study and the results (both experimental and numerical) in [35].

**Figure 7:**
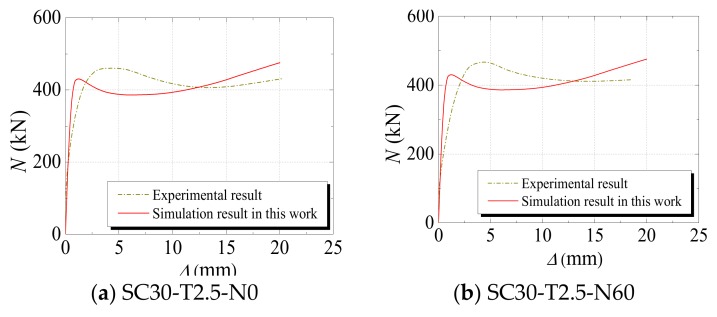

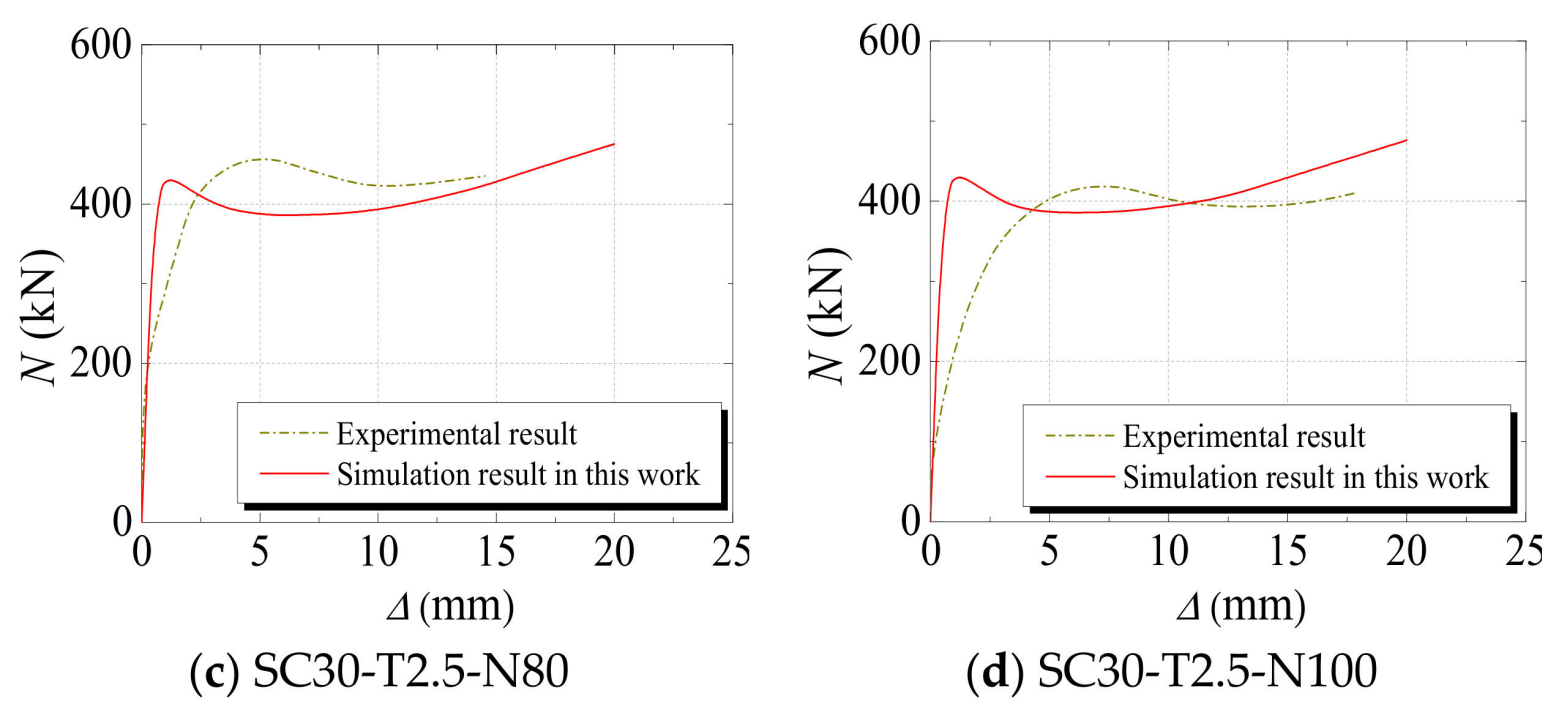
Comparisons between the numerical results in this study and the experimental results in [36].

Figure 5, Figure 6 and Figure 7 compare the axial load–displacement (or strain) curves between numerical and experimental results. In the figures, the dark yellow dot-dash line denotes the experimental result, the blue dotted line denotes the numerical result from the literature, and the red straight line denotes the numerical result from the FE model developed in this study. It can be observed that the increasing tendencies of the axial load–displacement (or strain) from the numerical results are generally consistent with those from experimental results. However, it is noted that the initial stiffness values of some specimens from the numerical results are different from those of the experimental results. The main reason for this phenomenon is that all the materials were assumed to be in the ideal condition in the numerical simulation. Additionally, measurement error may have resulted in a discrepancy of the initial stiffness. The specimens sometimes had different degrees of initial defects, which could also cause a deviation in the initial stiffness. Furthermore, the descending section of the curves indicates that, compared with the numerical results in the literature, the results calculated from the FE model developed in this study were closer to the experimental values: This sufficiently proves the ability of the proposed model to predict the axial load–displacement (or strain) curves of the specimens subjected to freeze–thaw cycles.

#### 2.3.2. Experimental Validation of the Acid Rain Corrosion Case

In this section, the experimental results for four specimens in [30] are used to verify the developed FE model’s performance in forecasting the ultimate bearing capacity of the specimen subjected to acid rain corrosion. The results of the comparison between the simulation and experimental values of the ultimate bearing capacity are shown in Table 2, in which *B* and *t* denote the side length of the specimen section and the initial thickness of the steel tube, respectively; ∆*t* denotes the reduction value of the wall thickness of the corroded steel tube; *γ* denotes the corrosion rate; *N_ue_* denotes the experimental value of the bearing capacity of the specimens; *N_ce_* denotes the corresponding numerical results in [30]; *N_be_* denotes the numerical results calculated by the developed FE model; and α denotes the initial steel ratio of the specimens. The mathematical expression of α is shown in Equation (32):

(32) $$\alpha =\frac{A_{s}}{A_{c}}$$

where *A_s_* and *A_c_* denote the cross-sectional areas of the steel tube and concrete in the initial state, respectively. The comparison results in the table show that the values of *N_ue_*/*N_be_* of all the specimens were less than 5%, which indicates the high prediction accuracy of the developed FE model. For each specimen, the experimental value is closer to the simulation result in this study than the simulation result in the literature. Therefore, it can be concluded that the established FE model is able to accurately predict the mechanical properties of SCFST specimens subjected to acid rain corrosion.

#### 2.3.3. Analysis of Validation Results

**Figure 8:**
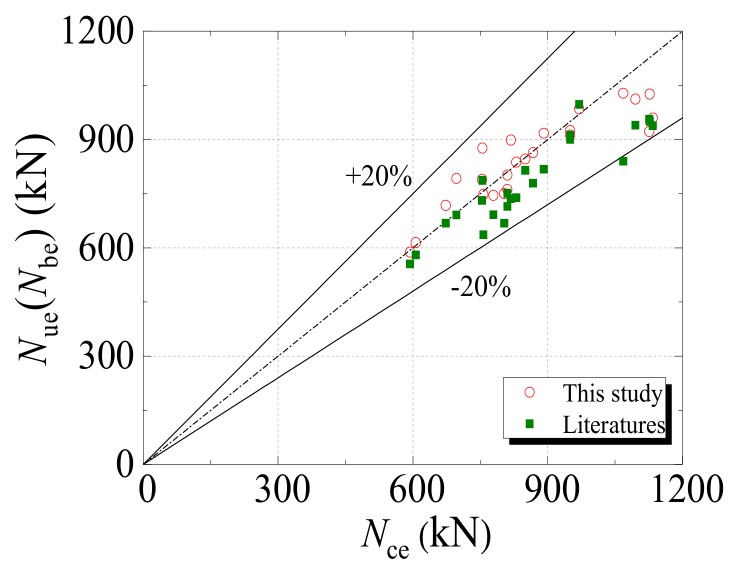
Comparisons between experimental and simulation results.

The numerical results for the bearing capacity in [24,30,35] were summarized and then compared with the experimental results, as well as the numerical results in this study. Figure 8 shows the comparison result, in which *N_ue_* and *N_be_* denote the simulation results for the bearing capacity from the literature and this study, respectively, and *N_ce_* denotes the corresponding experimental results. It is noted that, on the whole, the experimental results are closer to the simulation results of the model developed in this study than the numerical values from the relevant literature. Furthermore, the relative errors between the simulation results in this study and the experimental values were within 20%. Hence, the developed FE model is capable of characterizing the mechanical properties of SCFST stub columns under axial compression and the combined action of freeze–thaw cycles and acid rain corrosion.

## 3. Whole-Process Analysis of the Load–Displacement Relationship

### 3.1. Numerical Modeling for Axial Compression

The effect of the combined action of freeze–thaw cycles and acid rain corrosion on the bearing capacity of SCFST stub columns under axial compression was investigated by designing a set of FE models. The calculation parameters of the models are a cross-section width (*B*) of 400 mm, steel tube thickness (*t*) of 10 mm, column length (*L*) of 1200 mm, compressive strength of the concrete cube (*f_cu_*) of 40 MPa, yield strength of the steel tube (*f_y_*) of 235 MPa, Poisson’s ratios of concrete and steel of 0.2 and 0.3, respectively, and range of the combined frequency of freeze–thaw cycles and acid rain corrosion (*p*) of 1–5. In the combined process, it was assumed that the freeze–thaw cycles (*N_fn_* = 100) were performed first and that acid rain corrosion (*γ* = 10%) followed. This process was repeated to realize the combination of freeze–thaw cycles and acid rain corrosion. The detailed parameter values of the developed FE model are listed in Table 3.

### 3.2. Failure Mode

**Figure 9:**
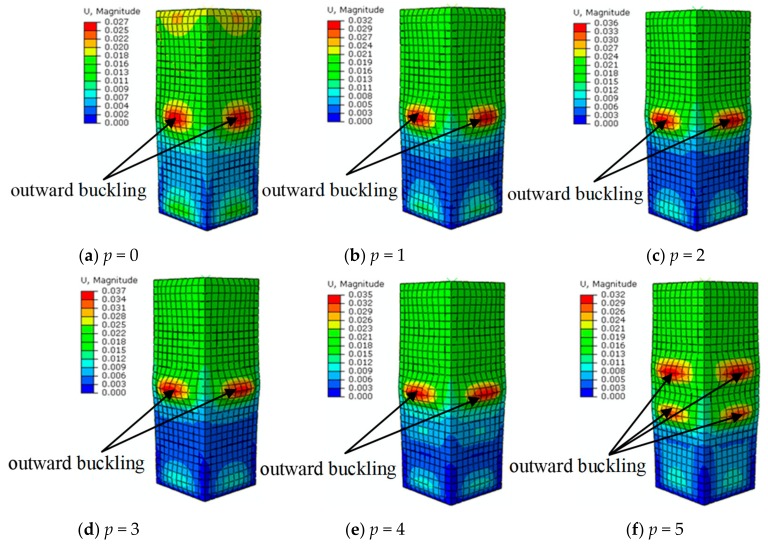
Failure modes of squared concrete-filled steel tube (SCFST) stub columns with different combined times of actions.

Figure 9 shows the numerical results for the failure mode of SCFST stub columns subjected to axial loading after suffering different cycles of freeze–thaw and acid rain corrosion. The figure shows that the failure mode of the columns was similar to that of an axially loaded column without any action. Buckling occurred at both ends and in the center of the column. In the real structures, the buckling directions of SCFST columns are all outward. The main reason for this phenomenon is that the inner core concrete of the stub column is capable of providing more effective support to the outer steel tube.

After the SCFST columns experienced different numbers of combined action, the columns were compared. The influence of combination times on the failure mode of the column can be summarized as follows: The larger the combination times, the more serious the local buckling of the steel tube. For the specimen, when the value of *p* gradually increased from 0 to 5, the peak buckling displacement increased from 0.027 to 0.037 m, which is a percentage increase of 37.04%. The peak value occurred when the value of *p* was 3. After that, the maximum buckling displacement decreased to 0.035 and 0.032 m, and the values of *p* were 4 and 5, respectively. Even though the buckling displacement decreased after the combined times exceeded 3, the buckling waves in the center part of the column increased from one to two, which is noticeable in Figure 9f. The main reason for this phenomenon is that the corrosion degree of the steel tube surface was almost the same because the corrosion of the specimens is uniform. When the combined number was small, the corrosion area of the specimen increased with the addition of combined times. Accordingly, there was an increased potential for local defects on the surface of the steel tubes induced by acid rain corrosion, together with the buckling degree at the defect location. Then, as the combined number continued to increase, the corrosion effect of the specimen became more serious, which decreased the effective thickness of the steel tube. Additionally, the buckling displacement also decreased when the steel tube was subjected to axial loading. Since the defect area was enlarged by the increasing corrosion effect, the number of buckling waves also increase. 

**Figure 10:**
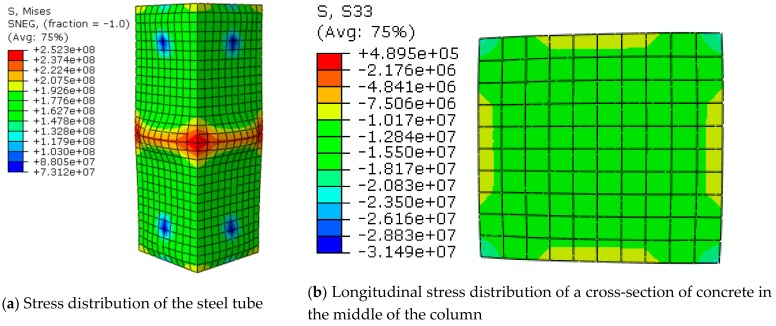
Stress distribution nephogram of steel tube and concrete.

Figure 10 shows the stress distribution nephogram of the steel tube and core concrete when specimen S-40-235-0.11-3 was axially loaded. Since the specimen was uniformly corroded, the stresses on the four surfaces were approximately the same. Hence, the buckling part of the steel tube and the damaged part of the core concrete were concentrated. For the steel tube, the maximum stress region could be found in the central section of the column, as shown in Figure 10a. The maximum stress at the corner was 252 MPa, which was due to stress concentration. This result indicates that the force for constraining the concrete was the largest at this location. Then, the stress of the steel tube decreased gradually from the central section to both ends along the column height. However, at the ends, the stress slightly increased, and the corresponding value was around 190 MPa. The main reason for this phenomenon is that external constraints in the end region of the column could cause the stress to increase. The longitudinal stress distribution nephogram in the cross-section of the column for the core concrete is depicted in Figure 10b. It is apparent that the maximum stress appeared at the corner of the column with a value of around 26 MPa, followed by the central area with a stress value of around 15 MPa. The minimum stress occurred in the center of the column edge with a value of about 10 MPa. This result indicates that the longitudinal stress distribution of the concrete in the center of the column changed as a result of the constraint effect of the external steel tube.

### 3.3. Load–Axial Displacement Relationship Curve

**Figure 11:**
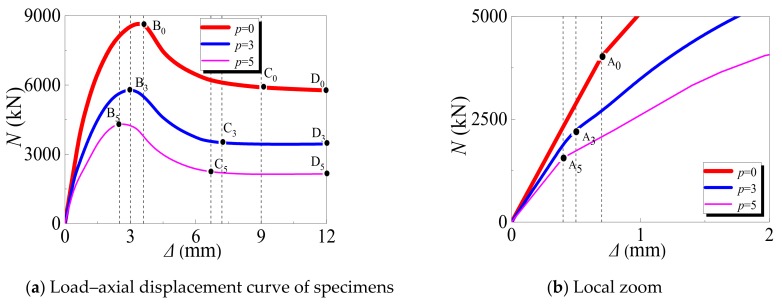
Load–axial displacement curve of specimens.

Figure 11 shows the relationship between the axial load and displacement of SCFST stub columns after their exposure to freeze–thaw cycles and acid rain corrosion. The combined times of the three curves O–A0–B0–C0–D0, O–A3–B3–C3–D3, and O–A5–B5–C5–D5 were *p* = 0 (*N_ft_* = 0, *γ* = 0), *p* = 3 (*N_ft_* =300, *γ* = 30%), and *p* = 5 (*N_ft_* = 550, *γ* = 50%), respectively. Overall, the increasing tendencies of the load–displacement curves corresponding to different combined times were almost the same. The stress process can be generally divided into four stages: The elastic stage (OA_i_), the elastic-plastic stage (A_i_B_i_), the descending stage (B_i_C_i_), and the flat stage (C_i_D_i_). A detailed analysis of each stage is reported below.

(1) Elastic Stage (OA_i_)

In the elastic stage, the relationship between the load and axial displacement is linear. Since the steel tube and concrete work independently, the interaction force between them is 0. Compared with the specimens under the normal condition (without any action, *p* = 0), the specimens that experience the combined action of freeze–thaw cycles and acid rain corrosion finish the elastic stage early. In addition, with the increase in combined times, the elastic modulus of the specimens gradually decreases, and the elastic phase ends increasingly earlier. This phenomenon is evident in Figure 11b: When *p* equals 0, the elastic stage is 0-A_0_; when *p* equals 3, the elastic stage is 0-A_3_; and when *p* equals 5, the elastic stage is 0-A_5_.

(2) Elastic-Plastic Stage (A_i_B_i_)

With the increase in axial load, microcracks in the concrete start to appear and continuously develop. At this stage, the transverse deformation coefficient value of the core concrete is larger than that of the steel tube, and the steel tube has transverse restraint on the core concrete. When the curve arrives at point B_i_, the specimens reach their ultimate bearing capacities. Compared with the specimens under the normal condition (without any action, *p* = 0), the specimens subjected to the combined action of freeze–thaw cycles and acid rain corrosion arrive at point B_i_ earlier. Furthermore, with the increase in the combination times, the ultimate bearing capacity of the specimen gradually decreases, and the time required to reach the ultimate bearing capacity decreases. When *p* equals 0, the corresponding ultimate bearing capacity is at point B_0_; when *p* equals 3, the corresponding ultimate bearing capacity is at point B_3_; and when *p* equals 5, the corresponding ultimate bearing capacity is at point B_5_.

(3) Descending Stage (B_i_C_i_)

After the specimens reach their ultimate bearing capacities, they cease working because of the crushing of the core concrete and the yielding of the steel tube. Finally, the specimens undergo plastic failure. Compared with the specimens under the normal condition (without any action, *p* = 0), the specimens that experience the combined action of freeze–thaw cycles and acid rain corrosion finish the descending stage earlier and then enter the stable phase. Moreover, with an increase in the combination times, the time required to reach the stable phase decreases. When *p* equals 0, the corresponding descending endpoint of the specimen is at point C_0_; when *p* equals 3, the corresponding descending endpoint of the specimen is at point C_3_; and when *p* equals 5, the corresponding descending endpoint of the specimen is at point C_5_.

(4) Stable Stage (C_i_D_i_)

Since the specimens are destroyed, the axial displacements of the specimens rapidly increase. However, regardless of whether the specimens undergo the combined action of freeze–thaw cycles and acid rain corrosion, they have a stable bearing capacity in the later stage, which indicates that the failures of the specimens are plastic failures.

### 3.4. Bearing Capacity

**Figure 12:**
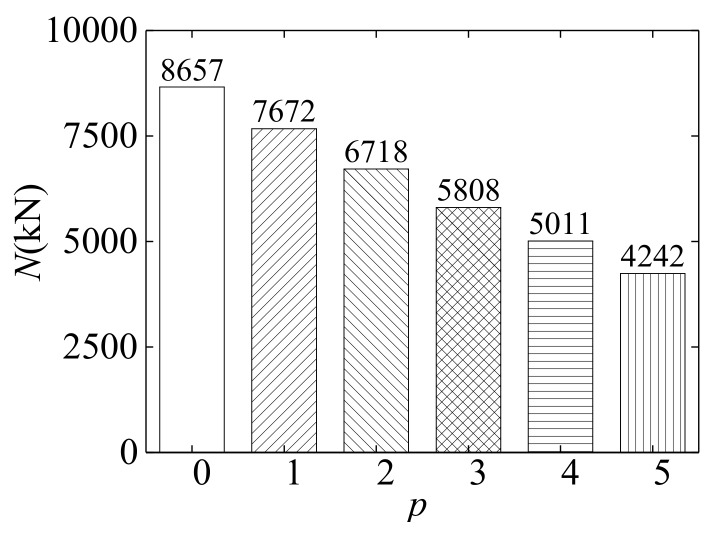
Influence of combined times on the ultimate bearing capacity of the specimen.

Figure 12 describes the ultimate bearing capacities of the specimens for different times of combined action of freeze–thaw cycles and acid rain corrosion. It is noted that the ultimate bearing capacity gradually decreased as the combined times increased. Compared with that of the specimen under the normal condition (*p* = 0), the ultimate bearing capacity of the specimen subjected to the combined action was reduced by 11.4%, 22.4%, 32.9%, 42.1%, and 51.0% when *p* equaled 1, 2, 3, 4, and 5, respectively. This result sufficiently shows that the combined actions of freeze–thaw cycles and acid rain corrosion have a significant influence on the ultimate bearing capacity of SCFST columns.

### 3.5. Interaction between the Steel Tube and Core Concrete

**Figure 13:**
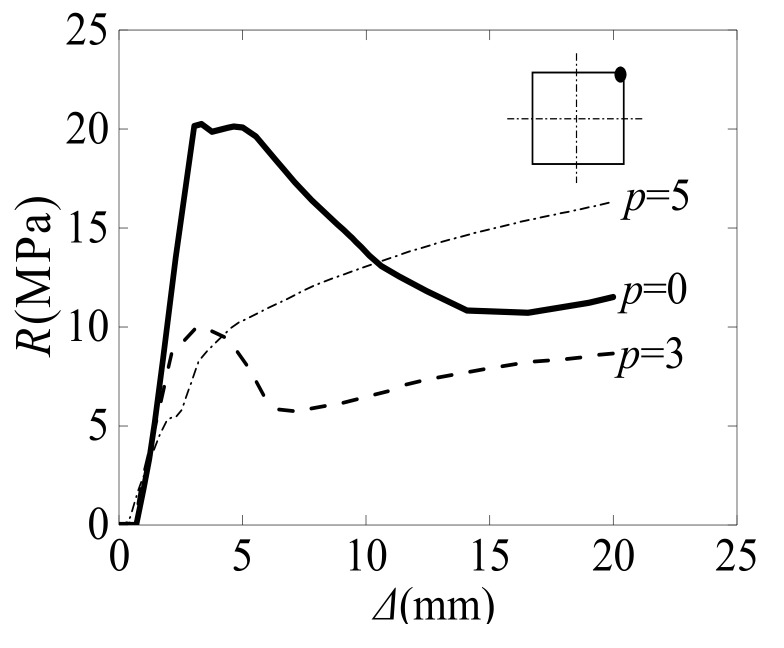
The relationship between the normal contact stress and axial displacement.

The normal contact stress (*R*)–axial displacement (*Δ*) relationship between the steel tube and the core concrete of the SCFST stub column is illustrated in Figure 13, in which the normal contact stress is measured at the corner of the section along the center of the column height. The figure indicates that when the axial displacement is initially applied, both the steel tube and core concrete are in the elastic working state. Because the Poisson’s ratio of the steel is larger than that of the concrete, the transverse deformation of the steel is larger than that of the concrete. Because of the gap between the steel tube and core concrete, the normal contact stress between them is zero. As the axial displacement continues to grow, the concrete begins to enter the elastic-plastic working state, and microcracks emerge on the surface of the concrete. In this case, the Poisson’s ratio of the concrete increases more quickly than that of the steel tube. When the transverse deformation of the concrete is larger than that of the steel tube, the normal contact stress between them is generated. With the increase in external load, the normal contact stress gradually rises. As the axial displacement continues to grow, the cracks in the core concrete continue to develop, and the specimen reaches its ultimate bearing capacity. At this time, the concrete surface is cracked and peeled off, the local buckling of the steel tube increases, and the normal contact stress gradually decreases. As the ultimate bearing capacity of the specimen gradually decreases, the axial displacement continues to increase, the core concrete is crushed, and the corresponding transverse deformation rapidly expands. At this point, the external steel enters the strengthening stage, and the restraint effect of the steel tube on the core concrete is enhanced. As a consequence, the normal contact stress between the steel tube and core concrete starts to increase. As observed in the figure, with the increase in combined times, the starting time of the interaction between the steel tube and core concrete becomes progressively earlier. The main reason for this phenomenon is that the more the core concrete is affected by the freeze–thaw cycles, the more the strength decreases, the greater the occurrence of surface cracks, and, thus, the earlier the specimen enters the plastic working stage. With the increase in combined times, the normal contact stress between the steel tube and concrete decreases gradually.

## 4. Parameter Analysis and Design Method of Bearing Capacity

According to [37], the parameter *K_s_* is defined as the influence factor of the bearing capacity of SCFST columns after axial compression and exposure to the combined action of freeze–thaw cycles and acid rain corrosion. The detailed mathematical expression is given in Equation (33).

(33) $$K_{s}=\frac{N_{n}}{N_{0}}$$

where *N_n_* denotes the ultimate bearing capacity of the specimen subjected to the combined action of freeze–thaw cycles and acid rain corrosion, and *N*_0_ denotes the ultimate bearing capacity of the specimen under the normal condition.

### 4.1. Parameter Analysis

In this section, the developed FE model is used to analyze the effects of the section size (*B*), steel ratio (*α*), cubic compressive strength of concrete (*f_cu_*), yield strength of the steel tube (*f_y_*), and combined times (*p*) on the influence factor *K_s_*. In order to facilitate the parameter analysis and propose the design method, it was assumed that a whole combination (*p*) consists of freeze–thaw cycles (*N*_ft_ = 100) and acid rain corrosion (*γ* = 10%). The mathematical relationship between *p*, *N*_ft_, and *γ* is provided in Equations (34) and (35). The basic parameters of the specimen were set to *B* × *t* × *L* = 400 × 10 × 1200 mm, *α* = 0.11, *f*_cu_ = 40 MPa, and *f*_y_ = 235 MPa. The ranges of the calculated parameters were set to *B* = 200–500 mm, *α* = 0.07–0.23, *f*_cu_ = 30–60 MPa, *f*_y_ = 235–420 MPa, and *p* = 0–5.

(34) $$\left[p,\;N_{ft},\;\gamma \right]=\left[\begin{array}{ccc} 0 & 0 & 0\\ 1 & 100 & 10\%\\ 2 & 200 & 20\%\\ 3 & 300 & 30\%\\ 4 & 400 & 40\%\\ 5 & 500 & 50\% \end{array}\right]$$

(35) $$p=0.00375N_{ft}\;+6.25\gamma$$

**Figure 14:**
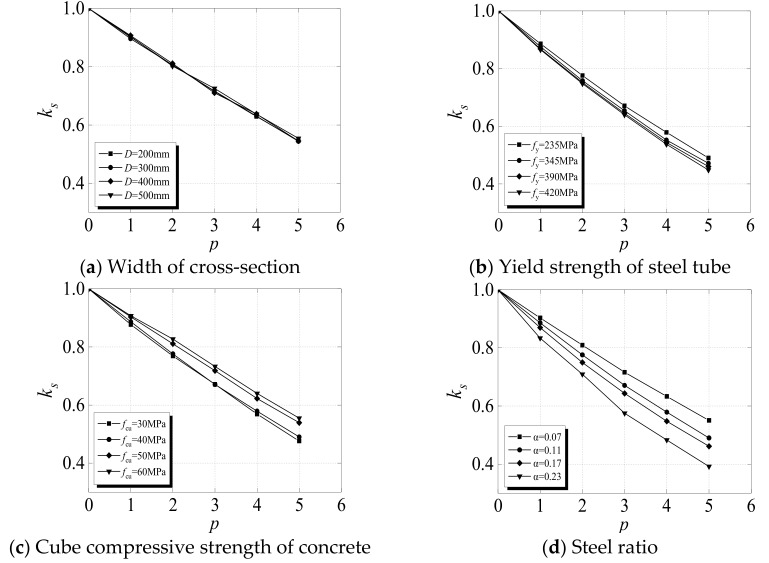
Influences of different parameters on *K_s_* (defined as the influence factor of the bearing capacity of SCFST columns after axial compression and exposure to the combined action of freeze–thaw cycles and acid rain corrosion) of the specimen.

Figure 14 portrays the influences of different parameters on the influence factor *K*_s_. As indicated by the four subfigures, the relationship between *K_s_* and the combination times (*p*) is approximately linear for all the parameters. With the increase in combination times, the value of *K*_s_ decreases, with different decreasing rates corresponding to different parameters. The maximum reduction in *K*_s_ can reach as high as 60%. Figure 14a shows the influence of section size (*B*) on *K_s_*. The four curves, which correspond to different values of *B*, almost overlap, which indicates that the width of the cross-section has little effect on *K*_s_. Figure 14b,c demonstrates the influences of the cubic compressive strength of concrete (*f_cu_*) and the yield strength of the steel tube (*f_y_*) on the value of *K_s_*, respectively. With the increase in combination times, the descending rates of the curves corresponding to different parameter values differ. Compared with *B*, *f*_cu_ and *f*_y_ have large influences on *K_s_* and can be considered the main factors. Notably, after experiencing the same combined times, the larger the value of *f*_y_ or the smaller the value of *f*_cu_, the smaller the value of *K*_s_. For instance, in the analysis range, when *p* equaled 3, the value of *f*_y_ increased from 235 to 420 MPa and the value of *K*_s_ decreased from 0.671 to 0.640. When the value of *f*_cu_ decreased from 60 to 30 MPa, the value of *K*_s_ decreased from 0.732 to 0.671. Figure 14d depicts the influence of the steel ratio (*α*) on *K*_s_. Similar to *f*_cu_ and *f*_y_, *α* has a significant influence on *K*_s_ and is also a major factor. In particular, for the same combination times, the higher the value of *α*, the faster the decrease in the value of *K*_s_. For example, when *p* equaled 3, the value of *α* increased from 0.07 to 0.23 and the value of *K_s_* decreased from 0.716 to 0.575. The main reason for this result is that the steel tube contributes more to the bearing capacity of the specimen than to the core concrete. Consequently, when the thickness and strength of the steel tube are reduced as a result of acid rain corrosion, the influence factor *K_s_* of the residual bearing capacity of the specimens significantly decreases. According to the definition of the constraint effect coefficient *ζ*, whose mathematical expression is given in Equation (36), if the values of *α* and *f*_y_ are larger and the value of *f*_cu_ is smaller, then the value of *K*_s_ is smaller. As a result, it can be concluded that the main factor that affects *K_s_* is *ζ*.

(36) $$\zeta =\alpha \cdot \frac{f_{y}}{f_{cu}}$$

### 4.2. Design Formulae

**Figure 15:**
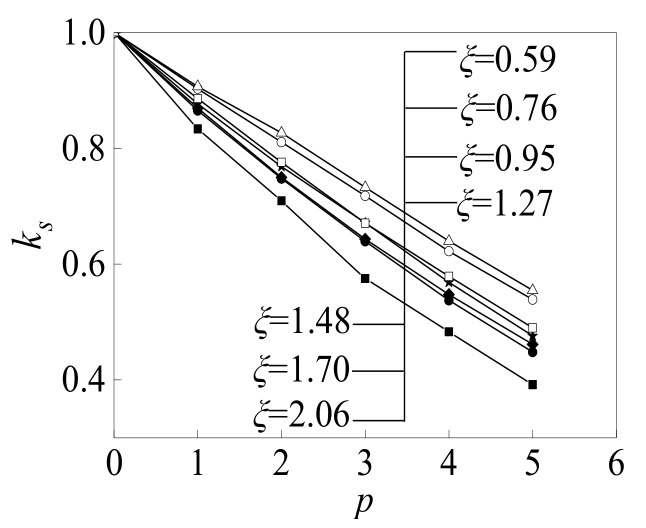
Influence of the constraint effect constraint effect coefficient *ζ* and combination times *p* on *K*_s_ of the specimen.

According to the parameter analysis result in Section 4.1, the parameters *ζ* and *p* are the main factors affecting *K*_s_. The influence of *ζ* on *K*_s_ is clearly illustrated in Figure 15, in which the value of *ζ* ranges from 0.59 to 2.06. Hence, the mathematical relationship between *K*_s_, *ζ*, and *p* can be obtained by using the regression method. According to the commonly used parameter ranges in a practical engineering application, the ranges of *ζ* and *p* are [0.5, 2] and [0, 5], respectively. Figure 15 shows that the relationships between *K*_s_ and *p* for different values of *ζ* are approximately linear. Accordingly, the following mathematical expression can be obtained:

(37) $$K_{s}=1+k\cdot p$$

where *k* is the function of *ζ*. From the numerical results, a simplified formula for *K_s_* can be obtained as follows.

(38) $$K_{s}=1.08-0.0667\zeta -0.105p$$

Currently, the specification GB50936-2014 [8] is commonly used to calculate the bearing capacity of SCFST stub columns in China. The formula for calculating the bearing capacity of SCFST stub columns under axial compression in the specification is given below:

*N*_0_ = (1.212 + *Dζ* + *Eζ*^2^) *A*_sc_*f_ck_* (39)

*B*_1_ = 0.131*f*_y_/213 + 0.723 (40)

*C* = −0.070*f*_ck_/14.4 + 0.026 (41)

where *A_sc_* denotes the combined cross-section area and *B*_1_ and *C* denote the restraint coefficients of the sectional shape. The substitution of Equations (38)–(41) into Equation (33) results in the equation for the residual bearing capacity *N_n_* of SCFST stub columns under the combined action of freeze–thaw cycles and acid rain corrosion, as shown in Equation (42):

*N*_n_ = (1.08 − 0.0667*ζ*−0.105*p*] · [1.212 + (0.131*f*_y_/213 + 0.723)*ζ* + (−0.070*f*_ck_/14.4 + 0.026)*ζ*^2^] *A*_sc_*f_ck_* (42)

where the ranges of the parameters *ζ*, *p*, *f*_y_, and *f*_ck_ are [0.5, 2], [0, 5], [0, 500], and [20, 40] MPa, respectively. 

**Figure 16:**
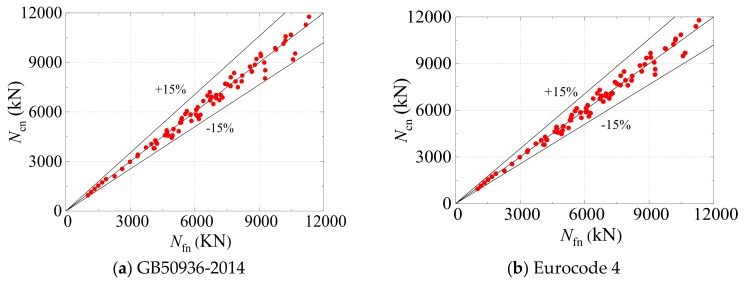
Comparisons between the simulation results from the finite element (FE) model and the calculation results from design formulae for calculating bearing capacity.

The accuracy of the proposed simplified design formula based on GB50936-2014 (Equation (42)) was verified by a comparison with the numerical results obtained from the FE model. The comparison is shown in Figure 16a, in which the results of simulating 72 SCFST stub columns under axial compression are compared with those calculated from the proposed design formula based on GB50936-2014 (Equation (42)). Minor discrepancies are observed between the numerical results from the FE model and the calculation results from the proposed design formula based on GB50936-2014. The mean value of *N_fn_*/*N_cn_* for 72 specimens was 1.014, and the corresponding standard deviation was 0.002. The relative errors could be controlled within ±15%.

In addition to the specification GB50936-2014, Eurocode 4 [11] also proposed a design formula for calculating the bearing capacity of a CFST member under axial compression, as provided in Equation (43):

*N*_0_ = *f*_y_*A*_s_+ *fc′ A*_c._ (43)

The substitution of Equations (38) and (43) into Equation (33) produces the equation for calculating the residual bearing capacity *N_n_* of SCFST stub columns under the combined action of freeze–thaw cycles and acid rain corrosion:

*N*_n_ = (1.08 − 0.0667*ζ*−0.105*p*) · (*f*_y_*A*_s_ + *f_c_′A*_c_) (44)

where the ranges of the parameters *ζ*, *p*, *f*_y_, and *f*_c_′ are [0.5, 2], [0, 5], [0, 500], and [24, 51] MPa, respectively. 

Similarly, the performance of the developed design formula based on Eurocode 4 was also validated using the numerical results. The evaluation result is shown in Figure 16b, in which the results of simulating 72 SCFST stub columns under axial compression are compared with the results calculated from the proposed design formula based on Eurocode 4 (Equation (44)). Compared with the design formula based on GB50936-2014, the formula based on Eurocode 4 performed better, with a mean value of *N_fn_*/*N_cn_* of 1.007 and a standard deviation of 0.002. Furthermore, the relative errors predicted by Equation (44) could also be controlled within 15%. 

Consequently, from the comparison results, it can be proven that both simplified design formulae are capable of predicting the residual bearing capacity of SCFST stub columns that are under axial compression and subjected to the combined action of freeze–thaw cycles and acid rain corrosion. Therefore, the outcome of this study can provide theoretical guidance for the safety evaluation, stability evaluation, and service life prediction of CFST structures in cold and acid rain areas.

## 5. Conclusions

This study numerically investigated the mechanical properties of the SCFST stub columns that are under axial compression and subjected to the combined action of freeze–thaw cycles and acid rain corrosion. An FE model was established using the software ABAQUS, and the model performance was validated using the experimental data captured from the literature. The effects of different model parameters on the residual capacity were studied, and design formulae were developed to evaluate the residual strength of SCFST stub columns in practice. The detailed conclusions are provided in this section.

(1) The failure mode of SCFST stub columns subjected to freeze–thaw cycles and acid rain corrosion is similar to that of columns without any action after they are axially loaded. Buckling occurs at both ends and in the central height of the columns. With the increase in the combined times, the local buckling amplitude of the steel tube increases.
(2) The load–displacement curves of SCFST stub columns under axial compression are basically the same after the columns are subjected to the combined action of freeze–thaw cycles and acid rain corrosion. All the curves include the elastic stage, elastic-plastic stage, descending stage, and stable stage. Under the same conditions, as the combination times increase, the times at which the specimens arrive at each stage are earlier. Regardless of whether the specimens undergo a combination of freeze–thaw cycles and acid rain corrosion, they all have stable bearing capacities in the later stage, and the failure type is a plastic failure.
(3) The parameters that influence the ultimate bearing capacity of the specimens after freeze–thaw cycles and acid rain corrosion include the restraint effect coefficient (*ζ_e_*) and combination times (*p*), which cannot be neglected. Generally, the larger the restraint effect coefficient or the combination times, the smaller the influence factor (*K*_s_) of the residual bearing capacity.
(4) From the result of parameter analysis, design formulae are proposed for predicting the bearing capacity of the SCFST column that is under axial compression and subjected to the combined action of freeze–thaw cycles and acid rain corrosion.

This paper reports a preliminary investigation into the effects of acid rain corrosion and freeze–thaw cycles on the mechanical properties of SCFST columns from a simplified perspective. Only uniform corrosion was considered in this study. In future work, the durability of CFST structures under local corrosion and freeze–thaw cycles will be analyzed. In addition, the mechanical properties of CFST structures due to other dual environmental factors and even multiple environmental factors will be studied for practical applications. The interdependency of multiple effects should be incorporated into the numerical analysis model to replicate the real-world situation. Related experiments will also be conducted in the laboratory, and the results will be used to further evaluate the effectiveness of the developed numerical model in predicting the mechanical properties of SCFST stub columns that are under axial compression and subjected to the combined action of freeze–thaw cycles and acid rain corrosion.

## Figures and Tables

**Table 1:** Bearing capacity comparisons between the numerical results and experimental results of specimens exposed to freeze–thaw cycles.

Specimen ID	*B* × *t* (mm)	*N_ue_* (kN)	*N_ce_* (kN)	*N_be_* (kN)	*N_ue_*/*N_ce_*	*N_ue_*/*N_be_*	Literature Sources
Sc1-50	100 × 2.06	604.5	618.3	646.4	0.977	0.934	[24]
Sc1-200	100 × 2.06	606.4	580.1	614.4	1.044	0.985	
Sc1-300	100 × 2.06	593.7	555.7	588.1	1.068	1.010	
Sc2-50	100 × 2.06	810.2	715.0	756.7	1.133	1.064	
Sc2-200	100 × 2.06	803.5	668.2	750.4	1.202	1.071	
Sc2-300	100 × 2.06	757.0	636.7	748.2	1.189	1.012	
Mean value					1.102	1.013	
Standard deviation					0.008	0.003	
S2-0	100 × 2	867.3	779.7	863.7	1.112	1.004	[35]
S2-50	100 × 2	810.3	751.7	801.6	1.078	1.001	
S2-100	100 × 2	753.9	731.1	789.7	1.031	0.955	
S2-200	100 × 2	779.1	692.9	745.4	1.124	1.045	
S2-300	100 × 2	673.0	668.6	717.8	1.007	0.938	
S3-0	100 × 3	891.3	818.3	917.6	1.089	0.971	
S3-50	100 × 3	818.0	736.0	898.4	1.111	0.911	
S3-100	100 × 3	754.7	787.2	876.4	0.959	0.861	
S3-200	100 × 3	830.0	739.2	837.4	1.123	0.991	
S3-300	100 × 3	696.9	691.1	792.2	1.008	0.880	
S3.9-0	100 × 3.9	1068.0	840.0	1028.5	1.271	1.038	
S3.9-50	100 × 3.9	1095.0	940.5	1011.9	1.164	1.082	
S3.9-100	100 × 3.9	1127.0	950.0	1026.2	1.186	1.098	
S3.9-200	100 × 3.9	1134.0	938.3	959.6	1.209	1.182	
S3.9-300	100×3.9	1126.0	956.3	922.3	1.177	1.221	
Mean value					1.110	1.013	
Standard deviation					0.007	0.011	
SC30-T2.5-N0	80 × 2.5	460.5		431.8		1.067	[36]
SC30-T2.5-N60	80 × 2.5	468.7		431.2		1.087	
SC30-T2.5-N80	80 × 2.5	458.2		430.9		1.063	
SC30-T2.5-N100	80 × 2.5	420.4		430.7		0.976	
Mean value						1.048	
Standard deviation						0.002	

**Table 2:** Bearing capacity comparisons between the numerical results and experimental results of specimens exposed to acid rain corrosion.

Specimen ID	*B* × *t* (mm)	∆*t* (mm)	*γ* (%)	*α*	*N_ue_* (kN)	*N_ce_* (kN)	*N_be_* (kN)	*N_ue_*/*N_ce_*	*N_ue_*/*N_be_*
S-114-0	114.00 × 2.97	0.00	0.0	0.113	950.0	912.0	921.2	1.042	1.031
S-114-1	113.42 × 2.97	0.24	9.6	0.102	970.0	997.0	985.0	0.973	0.985
S-114-2	112.78 × 2.97	0.51	20.5	0.089	950.0	900.1	902.5	1.055	1.041
S-114-3	112.20 × 2.97	0.76	30.2	0.078	850.0	814.7	846.3	1.043	1.004
Mean value								1.028	1.018
Standard deviation								0.002	0.001

**Table 3:** Main design parameters of the models.

Model ID	*B* × *L* × *t* (mm)	∆*t* (mm)	*f*_cu_ (Mpa)	*f*_y_ (Mpa)	*γ* (%)	*α*	*N*_fn_	*p*	*N* (kN)
S-40-235-0.11-0	400 × 1200 × 10	0	40	235	10	0.11	100	0	8657
S-40-235-0.11-1	400 × 1200 × 10	1	40	235	10	0.11	100	1	7672
S-40-235-0.11-2	400 × 1200 × 10	2	40	235	10	0.11	100	2	6718
S-40-235-0.11-3	400 × 1200 × 10	3	40	235	10	0.11	100	3	5808
S-40-235-0.11-4	400 × 1200 × 10	4	40	235	10	0.11	100	4	5011
S-40-235-0.11-5	400 × 1200 × 10	5	40	235	10	0.11	100	5	4242

Note: S means that the section of the specimen is square. The first number represents the compressive strength of concrete cubes. The second number represents the yield strength of the steel tube. The third number represents the initial steel ratio of the specimens (*α* = *A_s_*/*A_c_*, where *A_s_* and *A_c_* denote the areas of the steel tube and concrete in the initial state, respectively). The fourth number represents the combined times of freeze–thaw cycles and acid rain corrosion. For example, S-40-235-0.11-4 specifies square CFST specimens, compressive strength of concrete of 40 MPa, yield strength of the steel tube of 235 MPa, initial steel ratio of 0.11, and four alterations.
